# Evaluation of the Performance of Anganwadi Workers in Delivering Integrated Child Development Services in the Rural Field Practice Area of a Tertiary Medical College in South India

**DOI:** 10.7759/cureus.34079

**Published:** 2023-01-23

**Authors:** Ipsita Debata, TS Ranganath

**Affiliations:** 1 Community Medicine, Kalinga Institute of Medical Sciences, Bhubaneswar, IND; 2 Community Medicine, Bangalore Medical College and Research Institute, Bengaluru, IND

**Keywords:** preschool education, nutrition status, knowledge, growth monitoring, beneficiaries, icds, performance, integrated child development scheme, anganwadi workers, anganwadi centers

## Abstract

Background and objective

To meet the overall developmental needs of children below six years, adolescent girls, and expectant and nursing mothers, the Integrated Child Development Services (ICDS) was launched on 2nd October 1975 via the network of Anganwadis. It included a range of services like supplementary nutrition, preschool education, immunization, health check-up, referral services, and nutrition and health education. The majority of Karnataka's population resides in rural areas (61.3%) and among them, children (aged zero to six years) constitute around 12.05%. The incidence of mortality and morbidity among vulnerable groups is quite high. A total of 204 ICDS project areas have been sanctioned in Karnataka till 2012, out of which 63,377 Anganwadi centers (AWCs) from 185 projects have been functional. Findings show an alarming gap of 12 million beneficiaries for the required services.^ ^Mere 44% of children between 12 and 23 months have received complete immunization. The scheme has reached a stage where enriching its contents is more crucial than concentrating on universalization. Our study has tried to recognize the hindrances in the delivery of ICDS services by evaluating the performance of Anganwadi workers (AWWs), which in turn will optimize the benefit for the beneficiaries.

Materials and methods

This was a community-based, cross-sectional, descriptive study conducted over a period of six months. The tertiary medical college covers a population of 16,231 in its rural catchment area, whose health needs are served by 21 AWCs. All 21 centers were included in the study through the universal sampling method. Data collection commenced after obtaining clearance from the Institutional Ethics Committee and permission from the Program Officer and Child Development Project Officer (CDPO) of the concerned ICDS block. Informed consent was taken from all the AWWs. Data to evaluate the performance of AWWs were collected using a pre-validated and pre-structured questionnaire. Performance was evaluated by allotting scores to each question.

Results

Among the 21 AWWs interviewed, 38.1% belonged to the age group of 41-50 years. Of them, 76.2% achieved the correct number of target home visits per day. Growth charts were plotted correctly by the workers in only 60% of centers. Only 57.1% of centers stored raw food materials safely, away from infestation. In 71.4% of centers, the health staff had immunized the children appropriately. Only 38.1% of AWWs had the knowledge of giving paracetamol tablets to the mothers in case of fever and were also giving vitamin syrup to the children. Only 19% of AWWs responded correctly regarding the importance of Village Health and Nutrition Days (VHND). In our study, 11 (52.4%) workers had "good" knowledge about delivering different services under the ICDS scheme, eight (38.1%) had "poor" knowledge while two (9.5%) workers had "satisfactory" knowledge.

Conclusion

The present study gives some insight into the existing situation in rural AWCs. Although the majority of AWWs had good knowledge about delivering different services under the ICDS scheme, further improvement is needed for optimizing the outcome.

## Introduction

“Children are the world’s most valuable resource and its best hope for the future” - John F. Kennedy

As per the 2011 census, there are around 158.79 million children in India below the age of six years. Many children live in an underprivileged and deficient environment, which hampers their holistic development. Some of these conditions are poor sanitation, inadequate health care, poverty, and infections [1]. The government of India launched the National Policy on Children in August 1974 citing children as a “supremely important asset.” It also provided a framework for assigning importance to the different needs of the child. The Integrated Child Development Services (ICDS) was implemented on 2nd October 1975 as an initiative to cater to the needs of children below six years, adolescent girls, and expectant and nursing mothers [2]. The ICDS program is delivered through a network of projects in slums, rural, and tribal areas. The ICDS services are provided through a vast network of ICDS centers, better known as “Anganwadi.” The term Anganwadi developed from the idea that a good early child care and development center could be run with low-cost local materials even when located in an “angan” or courtyard. An Anganwadi center (AWC) is operated by a modestly paid Anganwadi worker (AWW), assisted by an Anganwadi helper or Sahayika. Each Anganwadi is supposed to cover a population of 1000 persons [3]. The type of services provided by the ICDS scheme through Anganwadis includes preschool education, health check-up, referral services, immunization, supplementary nutrition, and education on nutrition and health for children between three and six years [4]. The majority of Karnataka's population resides in rural areas (61.3%) [2] and amongst them, children (aged zero to six years) constitute around 12.05% [5]. The incidence of mortality and morbidity among vulnerable groups is quite high. A total of 204 ICDS project areas have been sanctioned in Karnataka till 2012, out of which 63,377 AWCs from 185 projects have been functional [6]. Findings show an alarming gap of 12 million beneficiaries for the required services [7]. The National Family Health Survey-5 found that a mere 44% of children between 12 and 23 months have received complete immunization, while more than half of the beneficiary children are undernourished. Iron and folic acid tablets are accessible to only 65% of women during pregnancy [8]. The scheme has reached a stage where enriching its contents is more crucial than concentrating on universalization. It is observed that even though ICDS has produced the changes, it is not uniform and not up to expectations. Our study has tried to recognize the quality in the delivery of ICDS services by evaluating the performance of AWWs, which in turn will optimize the benefit for the beneficiaries.

## Materials and methods

This was a community-based, cross-sectional, descriptive study conducted over a period of six months. The rural field practice under the tertiary medical college covers a population of 16,231 in its rural catchment area, whose health needs are served by 21 AWCs. All 21 centers were included in the study through the universal sampling method. Data collection commenced after obtaining clearance from Bangalore Medical College and Research Institute Ethics Committee (approval no: BMCRI/RP/EC/13/2013) and permission from the Program Officer and Child Development Project Officer (CDPO) of the concerned ICDS block. A pilot study was conducted at one of the AWCs in the designated rural area with the objective of standardizing the questionnaire and validating the scoring method. Informed consent for the study was obtained from all the AWWs. All AWWs of 21 AWCs were included. The nature and purpose of the study were explained to the AWWs. Data were collected by visiting the AWCs between 10 am and 2 pm and information was collected through direct observation and interview methods through a prestructured questionnaire. The basic information about AWWs was collected in terms of name, age, educational status, years of training, experience, etc., and the relevant data of AWWs regarding different aspects of services under ICDS provided at AWC were assessed by interview method. The data collected from interviews were also verified from records and available logistics at the center. A scoring system (pre-validated by a pilot study and developed arbitrarily) was used to assess the AWWs' knowledge about the correct way of delivering the different ICDS service components like supplementary nutrition (feeding support for 300 days a year), growth monitoring (children < three years weighed once a month and children aged three to six years weighed quarterly), pre-school education (adequate materials available for educating children under six years in a joyful and safe environment), immunization (beneficiaries immunized appropriately as per the National Immunization Schedule), health checkups (once every month by doctors from nearby health centers), and referral services (AWW enters names of beneficiaries requiring referral and refers them to the nearest health center at a timely manner) [3]. The sociodemographic profile of children was collected from the Anganwadi registers. The knowledge assessment score from each AWW was calculated based on the responses to the questionnaire containing 30 questions. The questionnaire was designed to contain questions on every aspect of services under the ICDS scheme, which was validated after the pilot study as mentioned earlier. One mark was given for a correct response, while no mark was given for a wrong response or unanswered question. The scoring system was developed for grading correct knowledge regarding the health service of each AWW. The health service knowledge of each AWW was scored out of 30. Workers with scores of <15, 15-20, and >20 were categorized as having poor, satisfactory, and good knowledge about delivering ICDS health services, respectively. Records were scrutinized for completeness, the number of registers, and recent updates.

## Results

The study was conducted among 21 AWCs. Each AWC covered around 676 people, five antenatal care (ANC) mothers or pregnant women, four lactating mothers, and three adolescents on average in the nearby area. An average of 35 children attended each AWC. Out of a total of 734 enrolled children, only 400 (54.5%) were present in the AWCs during the period of study. None of the pregnant or lactating mothers or adolescents were found to be present at the centers (Table 1).

**Table 1:** Quantitative assessment of the AWC AWC: Anganwadi center.

Parameter	Mean value ± SD
Number of people covered per AWC	676.05 ± 198.45
Number of children (0-6 years of age) enrolled per AWC	34.95 ± 9.23
Number of pregnant women enrolled per AWC	5.05 ± 1.02
Number of lactating women enrolled per AWC	4.05 ± 1.69
Number of adolescents enrolled per AWC	3.43 ± 5.20

Out of 21 AWWs, eight (38.1%) were in the age group of 41-50 years, seven (33.3%) were in the age group of 51-60 years, four (19%) were in the age group of 31-40 years, and two (9.5%) workers were more than 60 years of age (Table 2).

**Table 2:** Distribution of AWWs according to age AWWs: Anganwadi workers.

Age group (in years)	Frequency	Percentage
31-40	4	19.0
41-50	8	38.1
51-60	7	33.3
>60	2	9.5
Total	21	100

All 21 AWWs (100%) were found to have completed secondary school-level education. The maximum number of AWWs (16, 76.2%) had been working under ICDS for more than 10 years. Around 23.8% had an experience of five to 10 years (Table 3).

**Table 3:** Distribution of AWWs according to experience AWWs: Anganwadi workers.

Experience	Frequency	Percentage
5-10 years	5	23.8
>10 years	16	76.2
Total	21	100

All AWWs in our study had received induction and job orientation training at some point or the other after the appointment. Out of 21 workers, eight (38.1%) of them had undergone refresher training three to five times, mostly in Jayamahal, Bangalore. Seven (33.3%) of them had been trained more than five times while the remaining six (28.6%) had received training only one to two times to date (Table 4).

**Table 4:** Distribution of AWWs according to the number of refresher training sessions attended AWWs: Anganwadi workers.

Training sessions	Frequency	Percentage
1-2	6	28.6
3-5	8	38.1
>5	7	33.3
Total	21	100

In our study, it was found that 12 (57.1%) workers had attended a refresher training course more than one year ago while nine (42.9%) of them had attended the course within one year period (Table 5).

**Table 5:** Distribution of AWWs according to the duration of the last attended refresher training course AWWs: Anganwadi workers.

Years since the last training	Frequency	Percentage
≤1 year	9	42.9
>1 year	12	57.1
Total	21	100

Evaluation of the performance of AWW in delivering ICDS services

Growth Monitoring

In our study, out of 21 AWWs, 76.2% achieved the correct number of target home visits per day. Of the AWCs, 71.4% had maintained a growth chart for each beneficiary while 61.9% were monitoring growth from the birth of the child. In 66.7% of centers, children between three and six years of age were weighed every quarter while in 52.3% of centers, malnourished children were weighed every month. In 57.1% of centers, children under six months of age were weighed in an infant sling correctly. Growth charts were being plotted correctly by the workers in only 57.1% of centers (Table 6).

**Table 6:** Distribution of AWWs according to performance in components of growth monitoring * n = 21. AWWs: Anganwadi workers; AWC: Anganwadi center.

Components	Frequency of correct response^*^	Percentage
The target number of home visits by AWW	16	76.2
Each beneficiary child (birth-5 years+) of AWC has a growth chart	15	71.4
Growth monitoring status from birth	13	61.9
Children between 3 and 6 years are weighed every quarter	14	66.7
Children between 3 and 6 years who are malnourished are weighed every month	11	52.3
Children from birth to 6 months are weighed in an infant sling	12	57.1
Growth charts are plotted correctly: the information box is complete, weights are plotted to the nearest 100 g and joined to form a growth curve	12	57.1

Supplementary Nutrition Program (SNP)

All AWWs were preparing food as per the prescribed timetable. However, out of 21 AWCs, 90.5% prepared supplementary nutrition at a safe place (the kitchen was separate) but only 57.1% of centers stored raw food materials safely, away from infestation. Around 71.4% of centers responded correctly about the maximum number of feeding days per year and providing the prescribed quantity of supplementary food to the registered beneficiaries (Table 7).

**Table 7:** Distribution of AWWs according to performance in components of supplementary nutrition * n = 21. AWWs: Anganwadi workers.

Components	Frequency of correct response^*^	Percentage
Maximum number of feeding days as per the program	15	71.4
Supplementary nutrition meal is prepared at a safe place and the food is hygienic	19	90.5
The quantity of supplementary food given to each registered child and also to pregnant women and nursing mothers is as prescribed	15	71.4
The recipe is cooked as per the timetable (menu schedule)	21	100
The storage of raw food materials is safe (i.e., food is covered, dry, and free from infestation)	12	57.1

Immunization, Health Check-Ups, and Referral Services

In 71.4% of centers, the health staff had immunized the children appropriately, immunization records were up to date, health cards were maintained properly, the correct number of health checkups were being conducted by the health staff, and beneficiaries were being referred to the sub-center in case of any illness. However, in only 47.6% of centers, follow-up about health improvement in referred people was being done adequately. Only 38.1% of AWWs had the knowledge of giving paracetamol tablets to the mothers in case of fever and were also giving vitamin syrup to the children. In only 57.1% of centers, the AWW had prepared a list of beneficiary children according to their grades, i.e., grade normal, I, II, and III (Table 8).

**Table 8:** Distribution of AWWs according to performance in components of immunization, health checkups, and referrals * n = 21. AWWs: Anganwadi workers.

Components	Frequency of correct response^*^	Percentage
The health staff has immunized all those to be immunized	15	71.4
The mothers are given paracetamol tablets for controlling the rise in temperature	8	38.1
The immunization record is up to date	15	71.4
The health cards are maintained properly	15	71.4
Check from the diary of AWW the frequency of visits of health staff to the Anganwadi in the last 3 months for health check-ups of children	15	71.4
The AWW has prepared a list of beneficiary children according to their grades, i.e., normal, I, II, and III	12	57.1
The AWW has referred the beneficiaries to sub-center	15	71.4
The AWW follows the improvement in the health of those who have been referred	10	47.6

Nutrition and Health Education

Out of 21 AWWs, 57.1% of workers had conducted an adequate number of home visits for educational purposes per month and had prepared talking points to conduct nutritional education at the center. In only 47.6% of centers, adequate nutrition and health education sessions were organized every month, beneficiaries were mobilized to attend these sessions regularly, and the AWWs had prepared new health education aids (Table 9).

**Table 9:** Distribution of AWWs according to performance in components of nutrition and health education * n = 21. AWW: Anganwadi worker.

Components	Frequency of correct response^*^	Percentage
Target on number of home visits by AWW	12	57.1
The number of nutrition and health education sessions organized since last month?	10	47.6
The average number of women attending each session	10	47.6
AWW has prepared any talking points to conduct nutritional education	12	57.1
The AWW has prepared any new health education aids	10	47.6

Availability of Specific Services

All AWWs followed the fixed immunization day schedule, i.e., every Thursday. The beneficiaries were sent to the nearest health center for immunization. Poor responses were observed in other components related to the delivery of specific services under ICDS. Only 23.8% of AWWs conducted antenatal and under-five clinics regularly. Of the AWWs, 28.6% responded correctly about treating anemia in pregnant as well as non-pregnant women. Only 19% of AWWs responded correctly regarding the importance of Village Health and Nutrition Days (VHND) (Table 10).

**Table 10:** Distribution of AWWs according to components of availability of specific services * n = 21. AWWs: Anganwadi workers; VHNDs: Village Health and Nutrition Days.

Components	Frequency of correct response^*^	Percentage
Are antenatal clinics organized by Anganwadis regularly?	05	23.8
Are under-5 clinics organized by Anganwadis regularly?	05	23.8
Is the treatment of anemia given to both pregnant as well as non-pregnant women?	06	28.6
Is there a fixed immunization day?	21	100
Are VHNDs conducted?	04	19

Scoring of Performance of AWWs About Adequate Knowledge in Delivering ICDS

Based on the arbitrary scoring for performance evaluation of AWWs, it was observed in our study that the majority (11, 52.4%) of workers had "good" knowledge about delivering different services under the ICDS scheme, eight (38.1%) workers had "poor" knowledge while two (9.5%) workers had "satisfactory" knowledge (Table 11).

**Table 11:** Distribution of AWWs as per knowledge in delivering ICDS services AWWs: Anganwadi workers; ICDS: Integrated Child Development Services.

Knowledge performance score	Number of AWWs	Percentage of AWWs	Inference
<15	08	38.1	Poor
15-20	02	9.5	Satisfactory
>20	11	52.4	Good
Total	21	100	

Relationship Between the Performance of AWCs and its Factors

Assessment of the relationship between the knowledge performance of AWWs and different factors showed no significant association between the performance of AWWs and age of workers, years of service, number of training sessions attended, years since last trained, and finally presence or absence of an Anganwadi Sahayika (Table 12).

**Table 12:** Relationship between knowledge scoring of Anganwadi centers with different variables

Study variables	Performance score (n = 21)			χ2 (p)	95% CI
	Poor	Satisfactory	Good		
Age of Anganwadi workers					
≤55 years	5	1	10	2.89 (0.23)	0.19-0.21
>55 years	3	1	1		
Years of service					
≤10 years	1	1	3	1.39 (0.49)	0.61-0.63
>10 years	7	1	8		
Number of training sessions attended					
≤5	5	1	8	0.49 (0.78)	0.38-0.40
>5	3	1	3		
Years since last trained					
≤1 year	4	0	5	1.68 (0.42)	0.66-0.68
>1 year	4	2	6		
Presence of a Sahayika					
Yes	6	1	11	4.66 (0.09)	0.09-0.10
No	2	1	0		

## Discussion

Profile of Anganwadi workers

Each AWC covered around 676 people, five ANC mothers or pregnant women, four lactating mothers, and three adolescents on average in the nearby area. An average of 35 children attended each AWC. Out of a total of 734 enrolled children, only 400 (54.5%) were present in the AWCs during the period of study. None of the pregnant or lactating mothers or adolescents were found to be present at the centers. Similar findings were noted in a study by Thomas et al., where out of 826 registered children aged zero to six years, only 93 (11.3%) were attending the Anganwadi. None of the registered pregnant women, lactating mothers, and adolescent girls were present in any of the Anganwadis [9].

Out of 21 AWWs, eight (38.1%) were in the age group of 41-50 years, seven (33.3%) were in the age group of 51-60 years, and four (19%) were in the age group of 31-40 years. Two (9.5%) workers were more than 60 years of age. Similar findings were reported in a study by Madhavi et al., done in the Gulbarga district, where out of 15 AWWs, 46.6% of the AWWs belonged to the age group of 31-40 years, four (26.6%) were in the age group of 20-30 years, and two (13.3%) workers each were in the age group of 41-50 years and >50 years, respectively [10].

All 21 AWWs (100%) were found to have completed secondary school-level education. In a similar study done by Manhas and Dogra, out of 40 AWWs, 17.5% of the AWWs were under matric, 47.5% had passed the 10th standard, 27.5% had received higher secondary education, and 7.5% had completed graduation [11]. Another study conducted by Manzoor and Khurshid found that 70% of the workers were educated up to the 10th standard while 20% were educated up to the 12th standard and the rest of the workers (10%) were educated up to the middle level. It was found during the study that most of the workers were matriculates because the minimum educational eligibility for the post is the same and after getting the job they do not prefer to study further [12].

The maximum number of AWWs (16, 76.2%) had been working under ICDS for more than 10 years. Around 23.8% had an experience of five to 10 years. This finding corroborated with the results of a study conducted by Manzoor and Khurshid, where more than 50% of the workers had work experience of 10-20 years [12]. A study by Manhas and Dogra also found that the majority (65%) of the AWWs had work experience between 10 and 20 years, while 25% of them had an experience of less than 10 years [11].

All AWWs in our study had received induction and job orientation training at some point or the other after the appointment. Out of 21 workers, eight (38.1%) had undergone refresher training three to five times, mostly in Jayamahal, Bangalore, five (33.3%) had been trained more than five times while the remaining six (28.6%) had received training only one to two times till date. Better findings were reported in a study conducted by Dixit et al., where all 45 AWWs had received induction training, 36 (80%) of them had received job orientation training while 28 (62%) of them had received refresher training under ICDS [13]. A study by Manhas and Dogra found that out of 40 AWWs, the majority (92.5%) of AWWs were trained and had received in-service job training. Only 7.5% were untrained [11].

In our study, it was found that 12 (57.1%) workers had attended a refresher training course more than one year ago while nine (42.9%) had attended the course within one year period. A study conducted by Chaturvedi et al. found that, out of 80 AWWs, 65% had received a five-day refresher training in the past two years [14].

Growth monitoring

In our study, out of 21 AWWs, 76.2% achieved the correct number of target home visits per day. Of the AWCs, 71.4% had maintained a growth chart for each beneficiary while 61.9% were monitoring growth from the birth of the child. In 66.7% of centers, children between three and six years were weighed every quarter while in 52.3% of centers, malnourished children were weighed every month. In 57.1% of centers, children under six months of age were weighed in an infant sling correctly. Growth charts were being plotted correctly by the workers in only 60% of centers. In a study by Dixit et al., it was observed that growth charts were maintained in only 51% of AWCs and only 32.2% of workers were able to accurately plot and interpret the growth charts even though they all had been trained [13]. A study by Manhas and Dogra found that 25% of the AWWs had assessed the nutritional status of children in growth charts while 40% of the AWWs had assessed the nutritional status by weighing the child every month. It was also found that most of the AWWs (65%) were unaware of the importance of the growth chart and were only maintaining it because it was a job mandate. Of the AWWs, 15% said that they had to maintain growth charts only to report it to the parents of the registered children [11]. A study by Madhavi et al. showed very poor knowledge of growth monitoring. Out of 15 AWWs, only 16% responded correctly while the remaining 84% could not answer at all [10].

Supplementary nutrition

All AWWs were preparing food as per the prescribed timetable. However, out of 21 AWCs, 90.5% prepared supplementary nutrition at a safe place (the kitchen was separate) but only 57.1% of centers stored raw food materials safely, away from infestation. Around 71.4% of centers responded correctly about the maximum number of feeding days per year and providing the prescribed quantity of supplementary food to the registered beneficiaries. In 9.5% of the centers, hygiene was not followed properly, which exposed children to hazards of less space for activities, indoor air pollution, and gastrointestinal infections. This would affect the general development of children as a whole. A study by Sahoo et al. in Eastern Odisha found that dedicated cooking space for freshly cooked supplementary meals was present in only 16.7% of AWCs. The absence of a separate cooking place exposed the children to severe indoor air pollution [15]. Jain et al., in their study in Patiala, reported only 48.02% of beneficiaries received supplementary food from Anganwadis regularly. The irregularity was either because the AWC was not available or the Anganwadi was closed [16]. Similar findings were noted in a study by Thomas et al., who found that supplementary meals had been given during summer vacation. Supplementary nutrition was given to only three adolescent girls. Some AWWs were preparing food at their homes because of the absence of proper utensils at the centers [9]. Another study by Dixit et al. showed that cooking was done in the same classroom in the majority of the AWCs, which puts the children at great risk. The raw materials were also being stocked in the same room, which resulted in less space for conducting Anganwadi activities [13].

Immunization, health check-ups, and referral services

In 71.4% of centers, the health staff had immunized the children appropriately, immunization records were up to date, health cards were maintained properly, the correct number of health checkups were being conducted by the health staff, and beneficiaries were being referred to the sub-center in case of any illness. However, in only 47.6% of centers, follow-up about health improvement in referred people was being done adequately. Only 38.1% of AWWs had the knowledge of giving paracetamol tablets to the mothers in case of fever and were also giving vitamin syrup to the children. The remaining workers were afraid to prescribe drugs for minor ailments and referred the beneficiaries to the nearest health center. This might be because of a lack of proper knowledge about the use of basic drugs available at the centers. Proper training can help prevent the wastage of unused drugs as well as save the time of the beneficiaries. In only 57.1% of centers, the AWW had prepared a list of beneficiary children according to their grades, i.e., normal, I, II, and III. A study done by Dixit et al. found inadequate health checkups and vaccination services were being provided to the beneficiaries. There was a severe deficiency of medical kits and slips for a referral. Monthly health checkups were not being provided in all AWCs, which also affected the routine immunization schedules [13]. However, a study by Madhavi et al. assessed the knowledge of the AWWs regarding ICDS services and reported that 90% and 86.6% of AWWs had good knowledge about immunization and services related to referrals, respectively [10].

Nutrition and health education

Out of 21 AWWs, 57.1% of workers had conducted an adequate number of home visits for educational purposes per month and had prepared talking points to conduct nutritional education at the center. In only 47.6% of centers, adequate nutrition and health education sessions were organized every month, beneficiaries were mobilized to attend these sessions regularly, and the AWWs had prepared new health education aids. However, efforts must be made in the remaining centers to train the workers for the sake of the development of children and the benefit of other beneficiaries. A study by Jain et al. in Madhya Pradesh reported that AWWs spent only 9% of their time conducting home visits and a mere 3% of their time in growth monitoring. This warrants the identification of obstacles that are preventing the AWWs from devoting more time, which is a mandate by ICDS [17]. Inferior findings were reported in a study done by Manhas and Dogra, who said that only 50% of AWWs prepared charts pertaining to nutrition and health education, and only 15% were actually imparting education regarding nutrition without the aid of any educational materials. None of the AWCs followed the preschool timetable. Usage of slate and books were also limited. The children were mostly engaged with toys and no actual pre-formal education was being imparted [11].

Availability of specific services

All AWWs followed the fixed immunization day schedule, i.e., every Thursday. The beneficiaries were sent to the nearest health center for immunization. Poor responses were observed in other components related to the delivery of specific services under ICDS. Only 23.8% of AWWs conducted antenatal and under-five clinics regularly. Of the AWWs, 28.6% responded correctly about treating anemia in pregnant as well as non-pregnant women. Only 19% of AWWs responded correctly regarding the importance of VHND. This calls for urgent action. A study by Jha et al. in the Hoogly district of West Bengal identified that the major challenges faced by AWWs were inadequate logistics and lack of adequate manpower in conducting VHND [18]. Johri et al. conducted a study in the Hardoi district of Uttar Pradesh and reported that the cancellation of VHND sessions and non-attendance of beneficiaries rendered the objective of conducting VHND sessions futile [19]. A study by Dixit et al. found that local self-help groups were working in 19 AWCs and regular meetings with the ANMs and village health committees were being organized at only 11 centers [12].

Performance scoring of the AWWs

Based on the arbitrary scoring for performance evaluation of AWWs, it was observed in our study that the majority (11, 52.4%) of workers had "good" knowledge about delivering different services under the ICDS scheme, eight (38.1%) workers had "poor" knowledge while two (9.5%) workers had "satisfactory" knowledge. A similar performance study was done by Madhavi et al., in which out of 15 AWWs, 10 (66.67%) workers had "moderately adequate" knowledge, three (20%) had "inadequate" knowledge, and two (13.33%) had "satisfactory adequate" knowledge about delivering different services under the ICDS scheme [10]. Another study by Jena while estimating the correct knowledge score about ICDS schemes observed that the mean correct knowledge score is about 12.83 and the range varies from a minimum of seven to a maximum of 19 [20]. In a study conducted by Asha where the AWC was evaluated based on infrastructure facility, quality of preschool education, and supplementary feeding, out of 200 AWCs, 63.5% were efficient, 31.5% were not efficient, and only 5% were highly efficient [21]. Assessment of the relationship between the knowledge performance of AWWs and different factors showed no significant association between the performance of AWWs and the age of workers, years of service, number of training sessions attended, years since last trained, and finally presence or absence of an Anganwadi Sahayika. Similar findings were reported in a study done by Asha, where although a significant association was found between the efficiency of AWCs with education and job status of AWWs, no significance was found between the efficiency with age and years of service of AWWs [21].

## Conclusions

The present study gives us some insight into the existing situation in the rural AWCs. Although the majority of AWWs had good knowledge about delivering different services under the ICDS scheme, further improvement is needed for optimizing the outcome. Refresher training can be organized by the doctors affiliated with the tertiary hospital for the AWWs, in collaboration with the local administrative bodies, to improve their performance in delivering ICDS services, like timely referral and regular follow-up, conducting effective nutrition and health education sessions for beneficiaries, and planning more number of antenatal and under-five clinics in the AWWs, components which scored less in our study. The introduction of technology like mHealth by using mobile phones or computers in Anganwadis can be effective in the proper maintenance of data and also alert the AWWs to undertake necessary actions when pre-defined targets are not met. Other frontline workers like junior health assistants (JHAs), auxiliary nurse midwives (ANM), and Accredited Social Health Activists (ASHA) can be roped in to help the AWWs in monitoring the nutritional status of children by sending reminders about follow-up. Greater community participation will provide maximum benefits to the beneficiaries. A grievance redressal system can be set up by the administrative authorities to collect real-time feedback from AWWs regarding their limitations and requirements. Further qualitative research is warranted to understand the reasons behind the poor performance of some AWWs despite regular training courses by the government. These are some of the recommendations that can be considered.
